# Artificial Intelligence Chatbot Behavior Change Model for Designing Artificial Intelligence Chatbots to Promote Physical Activity and a Healthy Diet: Viewpoint

**DOI:** 10.2196/22845

**Published:** 2020-09-30

**Authors:** Jingwen Zhang, Yoo Jung Oh, Patrick Lange, Zhou Yu, Yoshimi Fukuoka

**Affiliations:** 1 Department of Communication University of California, Davis Davis, CA United States; 2 Department of Public Health Sciences University of California, Davis Davis, CA United States; 3 Department of Computer Science University of California, Davis Davis, CA United States; 4 Department of Physiological Nursing University of California, San Francisco San Francisco, CA United States

**Keywords:** chatbot, conversational agent, artificial intelligence, physical activity, diet, intervention, behavior change, natural language processing, communication

## Abstract

**Background:**

Chatbots empowered by artificial intelligence (AI) can increasingly engage in natural conversations and build relationships with users. Applying AI chatbots to lifestyle modification programs is one of the promising areas to develop cost-effective and feasible behavior interventions to promote physical activity and a healthy diet.

**Objective:**

The purposes of this perspective paper are to present a brief literature review of chatbot use in promoting physical activity and a healthy diet, describe the AI chatbot behavior change model our research team developed based on extensive interdisciplinary research, and discuss ethical principles and considerations.

**Methods:**

We conducted a preliminary search of studies reporting chatbots for improving physical activity and/or diet in four databases in July 2020. We summarized the characteristics of the chatbot studies and reviewed recent developments in human-AI communication research and innovations in natural language processing. Based on the identified gaps and opportunities, as well as our own clinical and research experience and findings, we propose an AI chatbot behavior change model.

**Results:**

Our review found a lack of understanding around theoretical guidance and practical recommendations on designing AI chatbots for lifestyle modification programs. The proposed AI chatbot behavior change model consists of the following four components to provide such guidance: (1) designing chatbot characteristics and understanding user background; (2) building relational capacity; (3) building persuasive conversational capacity; and (4) evaluating mechanisms and outcomes. The rationale and evidence supporting the design and evaluation choices for this model are presented in this paper.

**Conclusions:**

As AI chatbots become increasingly integrated into various digital communications, our proposed theoretical framework is the first step to conceptualize the scope of utilization in health behavior change domains and to synthesize all possible dimensions of chatbot features to inform intervention design and evaluation. There is a need for more interdisciplinary work to continue developing AI techniques to improve a chatbot’s relational and persuasive capacities to change physical activity and diet behaviors with strong ethical principles.

## Introduction

### Background

Physical inactivity and an unhealthy diet continue to be some of the leading risk factors for noncommunicable diseases (NCDs), such as cardiovascular disease, diabetes, and obesity [1,2], and death worldwide [3]. NCDs account for seven out of 10 deaths worldwide [3] and pose a substantial economic burden [4]. The prevalence of physical inactivity and an unhealthy diet varies considerably within and across countries. The United States is one of the countries experiencing a rapid rise in these risks. Nearly 80% of American adults do not meet the guidelines for both aerobic and muscle-strengthening activities [5], and the prevalence of overweight or obesity reached 71.6% in 2016 [6]. Therefore, developing cost-effective and feasible lifestyle interventions is urgently needed to reduce the prevalence [7].

Lifestyle modification programs have consistently evolved with emerging digital and communication technologies [8-13]. In the past two decades, there has been a large number of published studies using internet and mobile-based behavior interventions to support the effectiveness of using digital technologies to deliver intervention materials to diverse populations [8,14]. In recent years, the use of artificial intelligence (AI) and associated computational techniques has become the new frontier in expanding the landscape of health care and interventions [15].

### Definition and Applications of an AI Chatbot

AI chatbots, also called conversational agents, employ dialog systems to enable natural language conversations with users by means of speech, text, or both [16]. Conceptually, the core technical capacity of AI chatbots is different from that of embodied virtual conversational agents or avatars that emphasize on synthesizing multimodal signals (eg, images, videos, and sounds) to simulate human face-to-face communication. In this paper, we focused on developing the AI chatbot’s core feature of natural language conversation to facilitate more flexible information exchange between humans and the chatbot. The conversational capacity can range from constrained conversation (ie, users can only respond by selecting predefined conversational lines) to unconstrained conversation (ie, users can respond freely by inputting natural language conversational lines).

AI chatbots can be deployed in the form of mobile apps on smartphones, thus making programs available 24/7. AI chatbots have been rapidly transforming multiple fields, including business [17], governance [18], education [19], and health care [16,20]. As the top platforms supporting chatbot development, Amazon Alexa had more than 100,000 programs and Facebook Messenger had more than 300,000 active chatbots as of 2019, many of which are for health care and wellbeing. For instance, in April 2020, the World Health Organization launched a chatbot on Facebook Messenger to combat misinformation and to offer instant and accurate information about COVID-19 [21].

As chatbots increasingly become a convenient digital communication channel, they open up many opportunities for delivering personalized behavior change programs for disease prevention and health promotion on a large scale. Beyond connectivity and feasibility, the advantages of AI chatbot programs lie essentially in the computational power to develop and deliver personalized interventions [22-24]. Such interventions have the potential to overcome several limitations in the traditional paradigm of nonpersonalized interventions, as they are designed based on understanding individual characteristics and behavior trajectories and can incrementally adapt intervention strategies based on contextual conditions and personal cognitive and emotional states over time. In other words, chatbot technologies have the potential to “understand” individuals through natural human conversations, persuade individuals to change, and build sustaining supportive relationships for maintaining healthy behaviors.

### AI Chatbots for Health Care and Lifestyle Modification Programs

Chatbots for promoting physical activity and a healthy diet are designed to achieve behavior change goals, such as walking for certain times and/or distances and following healthy meal plans [25-29]. Although no systematic review of chatbots for lifestyle modification programs has been published, there are several reviews on chatbots covering health care issues ranging from mental health support and smoking cessation to disease diagnosis [16,30]. Owing to the different natures of targeted behaviors, some chatbots were mainly designed to provide information and knowledge [31], whereas others were developed based on established mental health intervention programs such as cognitive behavioral therapy [32]. One relevant review [33] focused on discussing the development of embodied conversational agents for a healthy lifestyle, and pointed out that the interpretation and application of behavior change theories were usually not reported.

Most previous chatbot research relied on either finite-state (ie, dialog consisting of a sequence of predetermined steps or states) or frame-based systems (ie, dialog is not predetermined but dependent on the content of the user’s input and the information that the system has to elicit) [34-36]. Such systems are restrained in their ability to allow free conversations, primarily due to the lack of large training data sets on human-to-human conversations in domains involving behavior changes.

The recent success of large pretrained language models, such as Bidirectional Encoder Representations from Transformers (BERT) developed by Google [37] and Generative Pre-Training-2 (GPT2) developed by Open AI [38], provides promising opportunities to incorporate language priors to down-stream natural language processing (NLP) tasks. For instance, several papers have shown that pretrained models can be tailored for task-oriented dialog generation, such as for conversations about restaurant recommendations and donation persuasion [39,40]. BERT and GPT2 are giant neural network models trained with large text data sets using self-supervised task objectives, such as recovering masked tokens and predicting the next word. As these models operate on representation space and do not have access to symbolic common-sense information, they produce outputs that are difficult for humans to interpret and can make errors that violate common senses in specific domains. One general direction to advance this field is to build systems that incorporate pretrained models to facilitate building dialogs that are specific for communicating and persuading users to adopt regular physical activity and a healthy diet.

To advance the science of developing effective and ethical AI chatbots for health behavior changes, especially within the context of improving physical activity and healthy eating behaviors, we provide a theoretical perspective and a model to guide the development and evaluation of AI chatbots for behavior changes. The aims of this perspective paper are threefold as follows: (1) to briefly summarize the current state of applications of AI chatbots in promoting physical activity and a healthy diet; (2) to propose the AI chatbot behavior change model developed by our research team; and (3) to address ethical considerations and principles.

## Methods

### Preliminary Review of AI Chatbot–Based Physical Activity and Diet Interventions

To provide a background of the current state of chatbot-based behavior interventions for physical activity and diet, we conducted a rapid preliminary literature review using four electronic databases (PubMed, EMBASE, Web of Science, and ACM Digital Library) on August 24, 2020. We used a combination of keywords to identify peer-reviewed studies related to AI chatbots for physical activity or diet (ie, [“chatbot” OR “conversational agent” OR “conversational system” OR “dialog system” OR “dialogue system” OR “relational agent”] AND [“physical activity” OR “exercise” OR “diet” OR “nutrition”]). We included only full-length articles that reported chatbot-based physical activity or diet interventions and were written in English. One researcher initially screened study titles and abstracts to determine eligibility for inclusion. Thereafter, two researchers reviewed the full texts of the included studies to further determine their relevance and coded study features. The two researchers discussed their disagreements throughout the coding process and agreed upon the final results.

In total, the search returned 108 articles from the four databases, with 15 published articles in 2020, 26 in 2019, 15 in 2018, 14 in 2017, five in 2016, and the remaining 33 from 2015 or before. After the screening, 101 (93.5%) articles were excluded for the following reasons: commentary or opinion pieces, scoping reviews, or empirical studies that addressed health domains other than physical activity and diet (eg, chatbots assisting diagnostic tasks or offering mental health interventions or treatment).

### Characteristics of AI Chatbot Interventions

We identified seven articles reporting six unique chatbots to increase physical activity and/or adoption of a healthy diet (Multimedia Appendix 1). Two papers reported on the same chatbot called Assistant to Lift your Level of activitY (Ally). One protocol [41] described the study design and one reported the actual optimization randomized controlled trial (RCT) (n=274) [42] for evaluating the effects of Ally in helping users to reach personalized daily step goals. The results showed that the intervention component of daily cash incentives delivered by Ally increased step-goal achievement. However, 30% of participants stopped using the app over the course of the study, presenting a challenge for the chatbot’s ability to engage participants. In contrast, another study reported the results of an RCT (n=106) [43] to evaluate the Healthy Lifestyle Coaching chatbot. The findings demonstrated that this chatbot was effective in increasing physical activity after 12 weeks of the intervention among office workers. The remaining four studies employed pretest-posttest designs. One feasibility study (n=23) [44] tested Tess, a behavioral coaching chatbot, in assisting adolescent patients to cope with weight management and prediabetes symptoms. Patients actively engaged with the chatbot, reported experiencing positive progress toward their goals, and deemed the chatbot helpful. One proof-of-concept study [45] reported on the Paola chatbot, which provided educational messages on physical activity and diet, weekly check-ins, and answers to user questions. The results showed that participants reported relevant weight loss and improved diet. Another validation study [46] reported on the CoachAI chatbot, which provided social and tailored health coaching support, and found this chatbot to be effective, especially among users with high engagement levels. Lastly, a chatbot named Reflection Companion delivered daily adaptive mini-dialogs and activity graphs to promote self-reflections. The conversations successfully triggered self-reflections that led to increased motivation, empowerment, and adoption of physical activity behaviors (eg, walking to a grocery store instead of taking a car) [26].

The above-reviewed chatbots showed preliminary evidence supporting the efficacy of using chatbots to deliver physical activity and diet interventions. It is worth noting that four out of seven (57.1%) studies reported chatbots as the only intervention used to deliver behavior change strategies [26,43,44,46], whereas the other three articles reported chatbots as an auxiliary component complementing other intervention approaches such as messages and conversations delivered by human facilitators [41,42,45] (Multimedia Appendix 1). The reviewed chatbots were designed with different theoretical components and varied in their abilities to engage in natural language conversations, relationship building, and emotional understanding. Overall, owing to a lack of reporting on the details of the theoretical framework and a limited number of RCT evaluations, it is difficult to systematically evaluate how different design theories and factors contribute to intervention efficacy. Based on this preliminary review, we identified a lack of systematic thinking in the development of AI chatbots for lifestyle behavior changes.

None of the studies reported in detail how they developed the chatbot program and none discussed ethical considerations regarding issues such as transparency, privacy, and potential algorithmic biases. Consequently, it remains unclear how to evaluate a chatbot’s efficacy, the theoretical mechanisms through which chatbot conversations influence users, and potential ethical problems. To address these gaps, in the next section, we present our theoretical framework that delineates design considerations, core theoretical components supporting a chatbot’s conversational capacity, multiple dimensions for usability and outcome evaluations, and ethical principles that need to be emphasized to guide development in this emerging field.

## Results

### AI Chatbots as Persuasive Technology

We conceptualize behavior change chatbots as a type of persuasive technology [14], which is more complicated than designing a social chatbot to engage in general conversations (eg, talking about movies or weather) [47]. Persuasive technology broadly refers to computer systems that are designed to change the attitudes and behaviors of users [48]. Behavior change chatbots thus aim to change users’ specific behaviors through engaging in conversations and delivering information and persuasive messages. In this regard, we propose that the chatbot dialog system needs to encompass two core capacities, including the relational capacity to establish and maintain a professional relationship with the user and the persuasive conversational capacity to change behaviors. Below, we describe a theoretical framework that elaborates on these two capacities and guides the design of AI chatbots for promoting physical activity and a healthy diet.

### Theoretical Framework: The AI Chatbot Behavior Change Model

**Figure 1:**
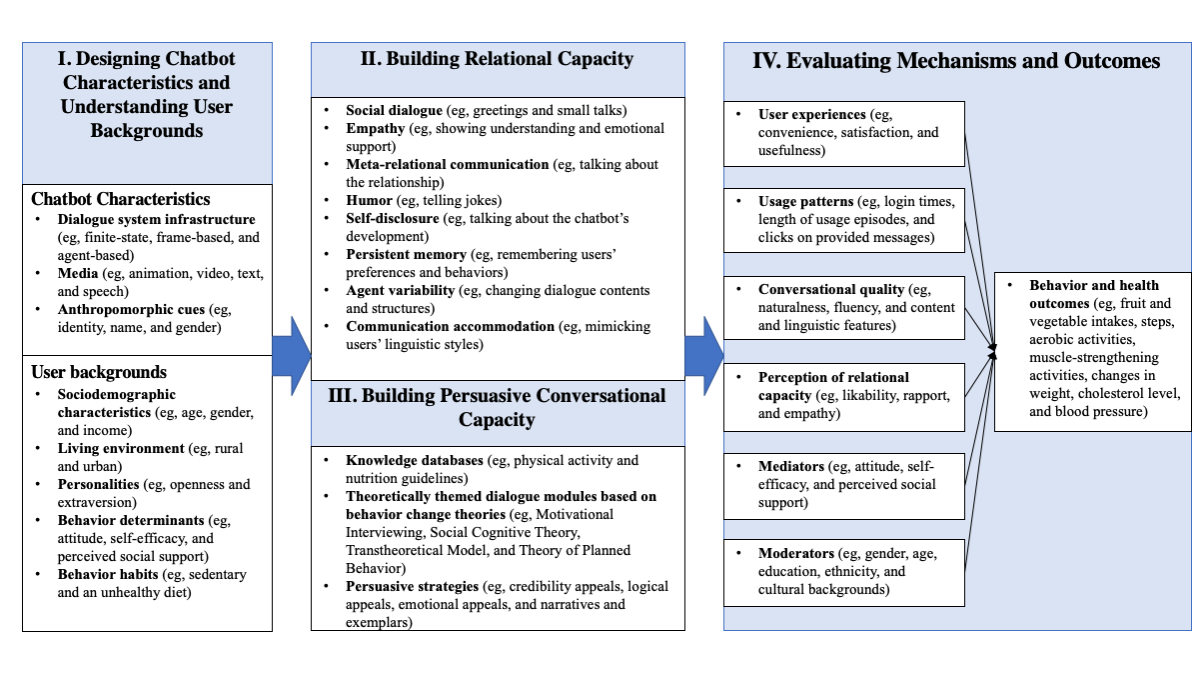
The artificial intelligence chatbot behavior change model.

Figure 1 shows the theoretical framework for improving physical activity and diet using AI chatbots. We named this framework the AI chatbot behavior change model, which includes the following four major components: (1) designing chatbot characteristics and understanding user backgrounds; (2) building relational capacity; (3) building persuasive conversational capacity; and (4) evaluating mechanisms and outcomes. The four high-level components are specified in sequence to guide the design and evaluation of chatbots. This proposed model is based on reviewing relevant chatbot studies, recent developments in human-AI communication research, and innovations in NLP, as well as our own clinical and research experience and findings [23,49-54].

#### Designing Chatbot Characteristics and Understanding User Background

Chatbots are set up to mimic the characteristics of human-human conversations. Designing a chatbot requires both system-related and agent-related considerations. Upon choosing a system infrastructure (eg, finite-state, frame-based, and agent-based infrastructure) and media (eg, animation, video, text, and speech), the characteristics of a chatbot (eg, identity, name, and gender) can be specified. In the past, researchers have experimented with using a robot [32], animal [55], or human identity, ranging in degrees of applying anthropomorphic cues [56].

The computers are social actors (CASA) paradigm [57] and the uncanny valley effect (UVE) [58,59] are the most widely used theoretical frameworks for studying human-computer interactions. While the CASA paradigm assumes that humans can develop positive social relations with computer systems as the human familiarity of the system increases, the UVE argues that too much human familiarity would bring feelings of eeriness and discomfort. To increase a chatbot’s social presence, some studies framed chatbots as peers and gave them gendered names (eg, Anna for female [27]). Deciding what name to call the chatbot and whether to frame it as a human peer or as a transparent bot system requires careful consideration. Our recent work [52] suggests that as AI chatbots are quickly adopting human conversational capacities, the perceived identity of a chatbot has significant effects on the persuasion outcome and interpersonal perceptions. Furthermore, our study findings suggest that users respond better if the chatbot’s identity is clearly presented. This may be because users can develop more agency and control if they know how to respond to the conversational partner by applying different communication norms. For instance, if a chatbot is presented with a human identity and tries to imitate human inquiries by asking personal questions, the UVE can be elicited and make people feel uncomfortable [52]. However, contrary findings have also been identified as some studies show evidence that people respond well and disclose more personal information if the chatbot is presented as a bot and can also display emotions [60,61]. Identifying the boundary conditions for chatbot identity and disclosures in various application contexts requires more research to provide empirical findings.

Designing a personalized chatbot system requires the understanding of each individual user’s background (eg, sociodemographic characteristics, living environment, and personality), behavior determinants, and habits [62-65]. The assumption is that a personalized intervention is more effective as it tailors both behavior change strategies and persuasive messaging to each user’s unique background and needs to achieve personally optimized outcomes [63]. In general, the first component serves to set up the chatbot characteristics and collect useful user background information to inform the development of algorithms supporting the second component and the third component. Theoretically, user background information can be incorporated as contextual information to develop algorithms to generate personalized relational messages and persuasive messages. Which characteristics can be used to tailor which messages depends largely on the target population’s needs and preferences [66,67]. Past literature has examined a number of useful characteristics for personalized influences, such as using different persuasive strategies to appeal to different personality traits [53,68] or setting personalized change goals based on behavior habits [42]. In the realm of physical activity chatbot interventions, the Ally chatbot system by Kramer et al was able to welcome each participant using personalized messages and track individual physical activity using the smartphone’s built-in accelerometer [42]. The system specifically set a personalized activity goal slightly above the participant’s current average activity level. Along this line, the application of control systems engineering in modeling individuals’ behavior states and adapting personalized goals over time is a promising approach [22].

#### Building Relational Capacity

In order to use an AI chatbot as a social conversational agent, we emphasize designing the system’s relational capacity in chatbot and user interactions [29,69-72]. Bickmore et al provided extensive discussions on the principles of building relational capacity in behavior change agents, such as using social dialog, empathy, meta-relational communication (talk about the relationship), humor, self-disclosure, persistent memory, and agent variability [70]. One of their studies showed that when compared to a nonrelational agent, a relational agent was more respected, liked, and trusted, which led to more positive behavior changes [29].

It is worth noting that most of the reported relational agents are embodied virtual agents, taking on specific anthropomorphic cues and nonverbal behaviors but using restricted scripted dialog designs. It remains less clear what relational capacity a nonembodied chatbot can achieve just through natural language conversations. Recent endeavors to accelerate natural conversations in everyday social companion chatbots have yielded promising results. One study reported that users of a companion chatbot (called “Replika”) perceived the chatbot to be human-like, intelligent, supportive, and able to facilitate social connection. However, UVEs also emerged as some users felt that the chatbot’s conversation was too natural and thus “creepy” [73]. In another case study that analyzed user reviews of the Amazon chatbot device, researchers found that over half of the reviewers referred to the chatbot using the personified name “Alexa,” and as users’ social interactions with the device increased, a greater level of personification occurred, which was associated with increased product satisfaction [74]. This suggests that people tend to personify Alexa, which is in line with the CASA paradigm. As a chatbot’s natural conversational abilities continue to rapidly improve, it is likely that relational capacity building can lead to better user engagement and retainment, despite other technological limitations.

To scale up the relational capacity in chatbots, conversational norms and relational strategies need to be built into the system. One approach can be through extracting patterns from longitudinal human-human conversations and drawing on theories from interpersonal communication and the latest human-AI communication research [75,76]. For example, the integrated model of advice giving [77,78] and the communication accommodation theory [79,80], combined with the chatbot’s capacity of persistent memory (eg, storing conversation history) and variability (eg, changing conversation content and structure), can provide useful insights in guiding the structure of conversations and specific choices in linguistic, semantic, and sentence styles.

#### Building Persuasive Conversational Capacity

Programs delivered by chatbots need to possess the core knowledge structures and intervention messages used in traditional approaches. Building behavior change messages into chatbot conversations first requires curating knowledge databases regarding physical activity and dietary guidelines. Thereafter, relevant behavior change theories need to be applied to generate themed dialog modules (eg, goal setting, motivating, and proving social support). Commonly used behavior change theories include motivational interviewing [81], the social cognitive theory [56], the transtheoretical model [82], and the theory of planned behavior [83]. One approach is to design human-human conversation episodes based on addressing each of the theoretical concepts (eg, a human interventionist providing social support to a participant) and to develop dialog modules that mimic such conversations.

In addition to delivering theory-based intervention messages, chatbots’ efficacy in eliciting behavior changes can be augmented by employing persuasive messaging strategies [84]. This thinking stems from the line of work in public health communication that aims to integrate behavior change theories and message effect theories (ie, theories that direct the selection of specific persuasive appeals and message features to enhance the effectiveness of communication) [85]. Persuasive strategies are designed to motivate behavior changes and are nuanced messaging choices to enhance attention, trust, and engagement, or to influence cognitive and emotional reactions. Persuasive strategies are important in shaping, changing, and reinforcing people’s attitudes and behaviors. Previous research has shown that even simply asking questions about a behavior can lead to changes in the behavior, known as the “question-behavior” effect. For instance, one study found that asking people questions about exercise led to an increase in self-reported exercise [86]. Although this effect was small and based on survey reports, it suggests that questions can function as a reminder or cue to action. Thus, one task of chatbots can be to ask questions to allow users to reflect and then get motivated for behavior change. More persuasive strategies can be embedded into theoretically themed dialog modules, such as using classical rhetorical appeals [53,68], including credibility appeals (eg, showing messages from sources that the target audiences trust), logical appeals (eg, providing reasoning and evidence for benefits of physical activity and a healthy diet), and emotional appeals (eg, using fear, guilt, or hope appeals for motivation). In addition, specific persuasive messaging strategies, such as using narratives and exemplars (eg, telling stories to enhance self-efficacy), can also enhance personal involvement and engagement. For example, to augment the approach of motivational interviewing, we can consider using credibility appeal to strengthen user’s trust in the chatbot, so that they become more comfortable in disclosing thoughts. In addition, to augment the approach of social cognitive theory, we can consider constructing narrative exemplars in terms of talking about relevant peers’ successful experiences to boost participants’ self-efficacy.

One common limitation of traditional programs is the static nature of persuasive messages, because of infrequent measurements of behaviors and users’ behavior change stages. Chatbots deployed on smartphones can address this limitation by utilizing ecological momentary assessment methods, in-built accelerometers, GPS, and other sensors, in addition to collecting user-reported data from convenient short surveys through the smartphone. For instance, research has shown that an accelerometer installed on smartphones is accurate for tracking step count [9] and that GPS signals can be used to estimate activity levels [87]. By objectively tracking and modeling activity patterns, developing machine learning models to update personalized goals and persuasive messages becomes feasible. Our work has shown that by using steps and physical activity intensity records, models can predict an individual’s probability of disengagement from the intervention [88]. Further, by using NLP and cluster analysis, we could differentiate individuals’ motivation levels as communicated in the conversation to tailor intervention maintenance programs [23]. These results indicate that AI chatbots can adapt not only behavior change goals and techniques, but also conversational styles (eg, emotional tones) based on learning from a user’s natural language inputs to enhance the engagement and effectiveness of messages.

Furthermore, rapid progress in mobile health technologies and functions has enabled the design of just-in-time adaptive interventions (JITAIs) [24]. JITAI designs in combination with real-time data from ecological momentary assessment, in-built accelerometers, GPS, and/or other sensors will allow chatbots to customize the timing, amount, content, and frequency of the intervention, by adapting each individual’s internal and external changes over time. However, a recent scoping review of health care chatbots showed that the use of JITAIs in designing and evaluating chatbots in health care in general and promoting physical activity and a healthy diet in particular is sparse, suggesting that future research needs to consider using more of these adaptive approaches [89].

#### Evaluating Mechanisms and Outcomes

Figure 1 shows the proposed dimensions for evaluating AI chatbot programs, including user experiences, usage patterns, conversational quality, perception of relational capacity, mediators, moderators, and behavior outcomes. All dimensions can be considered to improve the chatbot design and to understand theoretical mechanisms for how chatbot programs change behaviors.

User experiences concern users’ subjective evaluations of the overall interaction with the system. Many scales have been developed to assess a program’s convenience, satisfaction, usefulness, helpfulness, etc [90]. Usage patterns document objectively logged data regarding users’ interactions with the system, including records such as login times, length of usage episodes, and clicks on provided messages [91]. Conversational quality can be measured from users’ subjective evaluation of the conversation’s coherence, naturalness, and fluency. In addition, objective content and linguistic analyses of conversations can be used to assess specific dimensions of conversations such as the length of conversations and amount of information exchanged. Perception of relational capacity evaluates users’ perception of the chatbot identity and its relational capacity. Some studies have assessed the extent to which users deem a chatbot as a friend and its likability, as well as its capacity to achieve rapport, relate to human emotions, and show empathy [92-94]. Mediators refer to factors that help to explain why and how chatbot interventions are effective in promoting physical activity and a healthy diet. Chatbots can lead people to change their perceptions of themselves (eg, attitude, self-efficacy, and perceived social support) and help people to shape and form new behavior choices and patterns. These intermediate changes are important to explain the mechanisms of chatbot interventions and to design more effective interventions in the future. Moderators often refer to user characteristics such as gender, age, education, ethnicity, and cultural backgrounds, and these subgroups (eg, men vs women) may respond to a chatbot intervention differently. Advances in digital technologies can unintentionally reinforce or increase existing health disparities [95]. Thus, evaluating moderation effects is crucial in documenting a potential digital divide or lack thereof. Lastly, behavior outcomes denote actual changes in behavior and health, including diet (eg, fruit and vegetable intake five times per day [96]) and physical activity changes (eg, daily steps, aerobic activities, and muscle-strengthening activities [97]), and subsequent effects on health outcomes such as weight and blood pressure.

### Ethical Considerations

General ethical principles and guidelines for AI’s integration in health care need to be adopted in designing chatbots for lifestyle modification programs [15,98-100]. Key ethical considerations include having transparency and user trust, protecting user privacy, and minimizing biases. To gain the trust of users, credibility and transparency have to be established and communicated. A brief introduction of the intention and expertise of the research team behind the chatbot may enhance its credibility. Similarly, providing users with high-level explanations on the machine learning algorithms and data processing can help increase transparency. Protection of user privacy faces multiple challenges. There is emerging research showing that multiple sets of anonymized data can be modeled to reidentify individuals [101,102]. In the context of chatbot interventions, high standards of confidentiality and data anonymization, such as differential privacy [103], need to be adopted to decrease the risks of reidentification.

Within the context of persuasive health technology, beyond considering the general ethical principles in AI described above, another central framework that needs to be incorporated is the bioethics framework [104] consisting of (1) nonmaleficence, (2) beneficence, (3) respect for autonomy, and (4) justice. *Nonmaleficence* means the obligation to not inflict any harm or incur the least harm possible to reach a beneficial outcome. *Beneficence* denotes a moral obligation to act for others’ benefits. Building a commitment to nonmaleficence and beneficence means the chatbot’s intent is to benefit users with information, knowledge, care, and guidance, as well as to take positive steps to prevent and remove harm from the user. For example, chatbots need to be designed to understand expressions from users that indicate they may be undergoing difficult situations requiring human moderators’ help. Specifically, it is important to foresee and preemptively plan for the possibility that technical and algorithmic errors can occur, and it is pivotal to have human moderators in place to monitor user engagement regularly and be able to connect with users when challenging situations arise. *Respect for autonomy* means that the user has the capacity to act intentionally with understanding and without being controlled or manipulated by the chatbot. This specifies that users should be provided with full transparency about the intervention’s goals, methods, and potential risks. Given the complexity in AI and technological designs, researchers need to strive to provide comprehensible explanations that users can understand and then take decisions for themselves [105]. In addition, users should be fully informed in the consent process and consent form as to how their data will be used to improve the chatbot overtime during or even after the intervention and should be given the opportunity to opt out of having their data used in this manner. *Commitment to justice* requires researchers to consider the technology’s equity access and benefits to different populations, especially the consideration of high-needs users who are lower in socioeconomic status and digital literacy, or users with disabilities that could impact their interaction with chatbots. It is thus recommended that underserved populations, especially racial and ethnic minority groups, be represented and involved in all stages of the design and implementation of chatbot interventions to ensure health equity and social justice. Specifically, researchers need to consider applying debiasing strategies in building the dialog system [106,107] and socially aware algorithm design [108]. Given that the research field of using chatbots for behavior changes is still in its nascent phase, ensuring adherence to ethical principles and incorporating corresponding evaluative metrics is necessary for the field to move forward.

## Discussion

In this paper, we reviewed and synthesized literature involving lifestyle modification program studies, theories and studies from behavior science and communication research, and technical advancements in AI and NLP, and proposed the *AI chatbot behavior change model.* The strength of the proposed model is that it considers a wide range of chatbot-related components, including chatbot/user characteristics, relational capacity, and persuasive conversational capacity, and points out potential mediating and moderating factors to be evaluated to establish the efficacy of chatbots in changing physical activity and diet behaviors, as well as health outcomes.

To our knowledge, this is the first theoretical framework to provide a guideline to design and evaluate chatbot-based physical activity and diet behavior interventions. We contextualize the framework in the domains of physical activity and diet behaviors because these two are frequent daily behaviors that need continued engagement and monitoring. Chatbots as a convenient conversational tool can connect with people in real time to optimize behavior change interventions.

Moving science forward, systematic approaches and interdisciplinary collaborations are needed to design effective AI-based chatbot physical activity and healthy eating programs. Our proposed theoretical framework is the first step to conceptualize the scope of the work and to synthesize all possible dimensions of chatbot features to inform intervention design. However, when applied in specific contexts, researchers and practitioners can prioritize certain features that are mostly relevant to the target population, according to initial formative research conducted with the target population [54]. In essence, we encourage researchers to select and design chatbot features through working with the target communities using stakeholder-inclusive and participatory design approaches [109,110]. We think such inclusive approaches are much needed and can be more effective in bringing benefits while minimizing unexpected inconvenience and potential harms to the community. In this regard, we do not mean that every new chatbot program has to be developed from scratch. Previously established effective programs and their highlighted features can be incorporated and translated to a chatbot program and pilot tested with the target population. From there, the above-mentioned JITAI approach can be studied to test how different features can be adaptively applied to different individuals over time.

In summary, our study calls for more interdisciplinary work to continue enriching the conceptualization of a chatbot as a relational and persuasive agent and to develop approaches to leverage AI techniques to improve a chatbot’s relational and persuasive capacities with strong ethical principles. We call for future research to continue expanding and modifying this framework and to conduct empirical studies to evaluate its applicability in the actual design and assessment of interventions.

## Appendix

Multimedia Appendix 1 Summary of chatbot-based physical activity and diet interventions.

## Abbreviations

AI: artificial intelligence
BERT: Bidirectional Encoder Representations from Transformers
CASA: computers are social actors
GPT2: Generative Pre-Training-2
JITAI: just-in-time adaptive intervention
NCD: noncommunicable disease
NLP: natural language processing
RCT: randomized controlled trial
UVE: uncanny valley effect
