# Supplementary material for: Artificial Intelligence Chatbot Behavior Change Model for Designing Artificial Intelligence Chatbots to Promote Physical Activity and a Healthy Diet: Viewpoint
Source: J Med Internet Res. 2020 Sep 30;22(9):e22845. doi: 10.2196/22845 (PMC7557439; doi:10.2196/22845)
Supplement: Multimedia Appendix 1 [file jmir_v22i9e22845_app1.docx]

Supplementary Table 1. Summary of chatbot-based physical activity and diet interventions.

| **No.** | **First author/published year/Country** | **Study design** | **Theoretical framework and concepts** | **Sample size** | **Description of chatbot and intervention** | **Study findings** | **User input** | **Chatbot feature (only vs. auxiliary)** | **Ethical discussion** |
| --- | --- | --- | --- | --- | --- | --- | --- | --- | --- |
|  | **Title** |  |  | **Demographics** | **Duration of intervention** |  |  |  |  |
| 1 | Kramer JN/*  2020/  Switzerland | Optimization Randomized Controlled Trial (RCT) with 3 groups | Health action process approach | Cash or charity donation incentives intervention (n) = 186  No financial incentive control (n) = 88 | Chatbot:  Ally app delivered interventions via an interactive text-based chatbot interface and simultaneously collected contextual data using the smartphone’s built-in sensors.    Intervention:  Participants were randomized into 3 financial incentive conditions at baseline. In addition, they were randomized weekly to different planning conditions (action planning, coping planning or no planning) and self-monitoring conditions (receiving or not receiving a daily self-monitoring prompt). | Daily cash incentives increased step-goal achievement by 8.1% (95% confidence interval [CI]: 2.1 to 14.1).  Action planning increased step-goal achievement by 5.8% (95% CI: 1.2 to 10.4), only in the no-incentive control group.  Charity incentives, self-monitoring prompts, and coping planning did not result in physical activity increases (*p* > 0.005).  Engagement with planning interventions and self-monitoring prompts was low.  30% of the participants stopped using the app over the course of the study. | Constrained^†^ | Auxiliary | No |
|  | Which components of a smartphone walking app help users to reach personalized step goals? Results from an optimization trial [42] |  |  | Mean (SD) age:  41.7 (13.54)  Female: 57.7% | 8 weeks (2-week baseline, 6-week intervention) |  |  |  |  |
| 2 | Piao M/  2020/  South Korea | RCT with 2 groups | Habit formation model | Intervention (n) = 57  Control (n) = 49 | Chatbot:  Healthy Lifestyle Coaching Chatbot is a messenger app based on the habit formation model. It provided a health behavior intervention that emphasized the importance of sustainability and involvement.  Intervention:  The intervention group received cues and intrinsic and extrinsic rewards for the entire 12 weeks.  The control group did not receive intrinsic rewards for the first 4 weeks and only received all rewards as in the intervention group from the fifth to twelfth week.  The Self-Report Habit Index (SRHI) of participants was evaluated every week, and the level of physical activity was measured at the beginning and end of the trial. | After 4 weeks of intervention without providing the intrinsic rewards in the control group, the change in SRHI scores was 13.54 (SD ± 14.99) in the intervention group and 6.42 (SD ± 9.42) in the control group (*p* = .04).  When all rewards were given to both groups, from the fifth to twelfth week, the change in SRHI scores of the intervention and control groups was comparable at 12.08 (SD ± 10.87) and 15.88 (SD ± 13.29), respectively (*p* = .21).  The level of physical activity showed a significant difference between the groups after 12 weeks of intervention (*p* = .045). | Unconstrained | Only | No |
|  | Use of the healthy lifestyle coaching chatbot app to promote stair-climbing habits among office workers: Exploratory randomized controlled trial [43] |  |  | Age groups:  20s: 10.4%  30s: 51.9%  40s: 26.4%  50s: 10.4%  Female: 56.7% | 12 weeks |  |  |  |  |
| 3 | Maher CA/  2020/  Australia | Pre-and post-study | Goal-setting, problem-solving, goal review, self-monitoring with feedback, social support, reattribution, use of credible sources | N = 31 inactive community-dwelling adults | Chatbot: An artificially intelligent chatbot (Paola) guided participants through a computer-based individualized introductory session, weekly check-ins, goal setting, and was available 24/7 to answer questions.  Intervention: A 12-week MeLiPal program assisted users in increasing lifestyle physical activity and adopt a Mediterranean-style diet. The program incorporated behavior change techniques. Participants used a Garmin  Vivofit4 tracker to monitor daily steps, a website with educational materials and recipes, and a printed diet and activity log sheet. | After 12 weeks, Mediterranean diet scores increased from a mean of 3.4 (out of 14) at baseline to 9.6 (mean improvement was 5.7, 95% CI: 4.2 to 7.3).  After 12 weeks, participants lost an average 1.3kg (95% CI: -.01 to -2.5kg) and 2.1cm from their waist circumference (95% CI: -3.5 to -0.7cm). | Unconstrained | Auxiliary | No |
|  | A physical activity and diet program delivered by artificially intelligent virtual health coach: Proof-of-Concept study [45] |  |  | Mean (SD) age:  56.2 (8.0)  Female: 67.7% | 12 weeks |  |  |  |  |
| 4 | Stephens TN/  2019/  United States | Pre-and post-study | Cognitive behavioral therapy, emotionally focused therapy, behavioral activation, motivational interviewing | N = 23 youth with obesity | Chatbot:  A behavioral coaching chatbot (Tess) addressed different facets of behavioral health, such as depression and anxiety. Tess delivered customized integrative support, psychoeducation, and interventions through brief conversations via existing communication channels (i.e., SMS text messaging and Facebook Messenger).  Intervention:  The chatbot (Tess) was assessed for potential to improve the quality of care delivered in the existing weight management program. | The average duration of conversations between Tess and participants was about 12.5 (SD ± 15.6) minutes.  Adolescent patients reported experiencing positive progress toward their goals 81% of the time.  The 4,123 messages exchanged and patients’ reported usefulness ratings (96% of the time) illustrate that adolescents engaged with and viewed this chatbot as helpful. | Unconstrained | Only | No |
|  | Feasibility of pediatric obesity and prediabetes treatment support through Tess, the AI behavioral coaching chatbot [44] |  |  | Mean (SD) age:  15.2 (Not reported)  Female: 57.0% | Average 10-12 weeks |  |  |  |  |
| 5 | Fadhil A/  2019/  Not reported | Pre-and post-study | Health action process approach, technology acceptance model, AttrakDiff model | N = 19 individuals with sedentary lifestyle and poor dietary habit | Chatbot:  A conversational agent-assisted health coaching system (CoachAI) supported health intervention delivery to individuals.  Intervention: Participants interacted with the conversational agent daily. The goal of the study was to examine participants’ interactions with CoachAI and adherence to the instructions. Participants reported their overall experience with the conversational agent. | Users who were highly engaged were also more adherent to the activities delivered by the conversational agent. | Constrained | Only | No |
|  | Assistive conversational agent for health coaching: A validation study [46] |  |  | Mean (SD) age:  28.5 (9.4)  Female: 42.1% | 4 weeks (baseline – week 1, intervention – weeks 2, 3, 4) |  |  |  |  |
| 6 | Kocielnik R/  2018/  United States | Pre-and post-study | Structured reflection models | N = 33 active Fitbit users | Chatbot:  A mobile conversational system (Reflection Companion) supported engaging reflections on physical activity data collected with fitness trackers. Reflection Companion delivered daily adaptive mini-dialogues and graphs to users’ mobile phones to promote reflection.  Intervention: The study examined the system’s ability to engage users in reflection through dialogues with the conversational agent. Over the course of 2 weeks, participants received one mini-dialogue per day delivered to their mobile phones via SMS/MMS. | The mini-dialogues were successful in triggering reflection which led to increased motivation, empowerment, and adoption of new behaviors. | Unconstrained | Only | No |
|  | Reflection companion: A conversational system for engaging users in reflection on physical activity [26] |  |  | Mean (SD) age:  36.5 (11.2)  Female: 87.9% | 2 weeks |  |  |  |  |

Notes: * The protocol was published in 2019 [39].

^†^ Constrained conversation refers to conversation wherein the users can only respond by selecting pre-defined conversational lines; Unconstrained conversation refers to conversation wherein users can respond freely by inputting natural language conversational lines.
